# Barriers and facilitators of disclosing domestic violence to the healthcare service: A systematic review of qualitative research

**DOI:** 10.1111/hsc.13282

**Published:** 2021-01-13

**Authors:** Rebecca L. Heron, Maarten C. Eisma

**Affiliations:** ^1^ Department of Arts and Sciences University of Houston‐Victoria Victoria TX USA; ^2^ Department of Clinical Psychology and Experimental Psychopathology University of Groningen Groningen The Netherlands

**Keywords:** disclosure, domestic violence, healthcare personnel, healthcare service, qualitative research, systematic review, victims' experiences

## Abstract

Domestic violence victims are in frequent contact with the healthcare service yet rarely disclose. Therefore, it is critical to understand victims' experiences and perceptions regarding disclosure in healthcare settings. The goal of this review is to provide an updated synthesis of qualitative research identifying barriers and facilitators, advice, and positive and negative outcomes of adult victims' disclosure of domestic violence to healthcare professionals (HCPs). A systematic search of PsychINFO, CINAHL and Web of Science was conducted in January 2018. Thirty‐four eligible studies were identified, including 783 domestic violence victims (781 females). Formal quality assessment indicated variable study quality. Barriers of disclosure included negative HCPs attitudes, victims' perceptions of safety and concerns about the consequences of disclosing. Facilitators of disclosing included a positive relationship with the HCP, HCPs directly asking victims about abuse, and HCPs ensuring that the environment is safe and disclosure is confidential. Victims advised increased awareness of HCPs reactions to disclosure and avoiding mirroring their perpetrators minimization. HCPs were encouraged to engage in direct questioning and maintain a supportive and secure environment. Positive and negative outcomes of abuse were identified, such as being able to leave the abuser or, on the other hand, the victims' situation not changing. Our results indicate that barriers for disclosure of domestic violence in healthcare settings persist despite the widespread implementation of policies and guidelines to counter them. Based on these findings, we provide recommendations for clinical practice and future research to help improve disclosure in healthcare settings.

**What is known about this topic?**

- Domestic violence victims are in frequent contact with healthcare professionals, yet rarely disclose.
- Disclosure can help victims become free from violence.
- International guidelines exist to facilitate disclosure in healthcare settings

**What this paper adds?**

- Our up‐to‐date synthesis of qualitative research identified a variety of barriers and facilitators of disclosure, victim advice on disclosure, and outcomes of disclosure in healthcare settings.
- Barriers and facilitators identified in previous studies persisted, yet we also identified new barriers and facilitators.
- Healthcare professionals are encouraged to engage in direct questioning and maintain a supportive and safe environment. Positive outcomes of disclosure included feeling validated and leaving the abuser. Negative outcomes included the stigmatization and continued abuse.

## 1 INTRODUCTION

Domestic violence, also referred to as intimate partner violence, is a large public health problem in the UK and worldwide (Campbell et al., 2002; Hegarty et al., 2004; World Health Organization, 2013a). According to the Department of Health (2017) one in four women and one in six men in England and Wales suffer domestic violence in some form. Domestic violence refers to controlling, threatening, or coercive behaviour, violence or abuse between those aged 16 or older who are or have been intimate partners or family members. This includes psychological, physical, sexual, financial and emotional types of abuse (Home Office, 2013).

Victims of domestic violence commonly experience a variety of physical and mental health problems because of abuse (Diaz‐Olavarrieta et al., 2009; Hegarty, 2011; Hindin et al., 2008). Physical health problems include sexually transmitted infections, pelvic inflammatory diseases and menstrual irregularities (Plichta & Abraham, 1996; Schei & Bakketeig, 1989). Psychological health problems include post‐traumatic stress disorder, depression, anxiety, low self‐esteem, psychosomatic complaints, increased substance abuse, self‐harm and suicidal ideation (Bergman et al., 1987; Rounsaville and Weissman, 1977–1978). Consequently, abused women are much more likely to be in contact with the healthcare service than non‐abused women (Garcia‐Moreno et al., 2015) and are more likely to be in contact with the healthcare service than any other professional service (Feder et al., 2006). The healthcare service therefore appears uniquely positioned to prevent and intervene in domestic violence.

Accordingly, policies and regulations to improve healthcare services identification and responses to disclosures from victims of domestic violence have been introduced (Department of Health, 2000; World Health Organisation, 2013b). Direct questioning is an effective strategy facilitating domestic violence victims' disclosures (Cann et al., 2001; Howard et al., 2010). Disclosure can help victims to become free from violence within 6 weeks in situations when they are given appropriate care following disclosure (Krasnoff & Moscati, 2002).

Yet, despite healthcare providers (HCPs) significant advantage in accessing this hard to reach population and the potential benefits of disclosure, HCPs have not always been effective in identifying victims of domestic violence (Campbell et al., 2002; Chapman & Monk, 2015; Feder et al., 2011). Only 10%–50% of the domestic violence cases are detected in healthcare services (Feder et al., 2006; Gremillion, & Kanof, 1996). A barrier to disclosure may be that HCPs do not feel capable or comfortable discussing domestic abuse (Taylor et al., 2013). Additionally, HCPs have reported that a lack of time, privacy, training, resources and knowledge on how to ask about domestic violence have prevented them from enquiring about abuse (Beynon et al., 2012; Sundborg et al., 2012). For example, doctors and nurses report receiving little or no training in responding to domestic violence (Rimmer, 2017; Taft et al., 2004). Perhaps unsurprisingly, victims have reported that HCPs have been inappropriate, inadequate and unhelpful in responses to disclosures of abuse (Pratt‐Erickson et al., 2014; Trevillion et al., 2011, 2014).

To improve responses from HCPs, it is also important to understand how victims perceive and experience disclosure in healthcare settings. More than a decade ago, a systematic review of qualitative studies by Robinson and Spilsbury (2008) on disclosing domestic abuse to HCPs first summarized findings on this topic. They charted what barriers and facilitators of disclosure domestic violence victims reported. They found that victims wanted the topic of abuse to be routinely raised by HCPs to make it easier to disclose, yet also had concerns about disclosing. For example, victims felt that just one consultation with a professional was not enough to build the trust needed to disclose and that the brevity of appointments with their HCP limited opportunities for disclosure. Victims also reported that a lack of privacy at their healthcare setting prevented them from disclosing. Other major barriers were victims' fears that they would lose their children or that the abuse would escalate.

Robinson and Spilsbury's (2008) review focussed on the barriers and facilitators that victims experience and perceive when disclosing abuse to the health service, yet they did not look specifically at victims' advice provided to HCPs or their reported outcomes of disclosure. It appears important to also summarize such advice as victims themselves could be viewed as “experts” of their own experiences enabling them to bring unique insight and knowledge (Reid et al., 2005) which may improve future victims' experiences of disclosing abuse.

Additionally, it appears critical to investigate victims' reported outcomes of disclosure. For example, a positive outcome might be a victim experiencing direct change after disclosing such as them leaving their partner or filing a police report (Liebschutz et al., 2008), whereas a negative outcome of disclosure could be the victim feeling responsible for the abuse and becoming convinced that nothing will change (Damra et al., 2015). Charting such outcomes could provide further insight into the potential benefits or drawbacks of disclosing which in turn could help motivate HPCs to act in line with established guidelines and regulations to facilitate disclosure. The current review therefore sought to update and extend Robinson and Spilsbury's (2008) review, to provide a more comprehensive, up‐to‐date overview of qualitative research of victims' views and experiences related to disclosure.

### 1.1 Aim of the review

While Robinson and Spilsbury (2008) provided an important starting point to better understand barriers and facilitators of disclosure, their review was limited in size (including 10 papers), scope (focusing only on barriers and facilitators and including only studies from English‐speaking samples) and rigour (study quality was not systematically assessed). To overcome these limitations, we conducted an updated systematic review of qualitative studies that investigated experiences and perceptions of domestic violence victims on disclosure in healthcare settings. This review includes a decade of new research on barriers and facilitators, expands the scope of the review to include victims' advice and outcomes of disclosure, and weighs the evidence by providing a systematic assessment of study quality.

Specifically, the review aimed to address the following questions:

1. What barriers and facilitators do victims of domestic violence experience or perceive when disclosing abuse to the healthcare service?
2. What advice do victims give on ways that the healthcare service can increase victims' disclosures of domestic violence?
3. What are the outcomes of disclosing domestic abuse to health professionals? (positive or negative, e.g., was the victim able to leave the abusive relationship?)

## 2 METHOD

This review was conducted in agreement with the guidelines and criteria for systematic reviews reporting set out by the Preferred Reporting Items for Systematic Reviews and Meta‐Analyses (PRISMA) statement (Moher et al., 2009). This review was not pre‐registered as the preparation for this review started before PRISMA was widely applied.

### 2.1 Search strategy

A comprehensive search of three electronic databases, PsychINFO, CINAHL and Web of Science, was conducted on the 30 January 2018 (see Appendix S1). The keywords used in the search strategy were drawn from the previous systematic review conducted by Robinson and Spilsbury (2008). Additional search terms were added by checking the keywords used in articles identified in preliminary searches. A Boolean approach was used, and the following search terms were chosen after scoping the literature: ["Domestic* violence*" OR "battered female*" OR "intimate partner violence” OR “partner abuse*” OR “domestic abuse*” OR “battered men*” OR “battered male**” OR “battered women*” OR “victim*” OR “spouse abuse*” OR “survivor*” OR “female survivor*” OR “male survivor*” OR “intimate partner violence survivor*”] AND [“Self‐Disclosure” OR "Disclosure*" OR "help seeking"] AND [“Health care service*”OR “Health care professional*” OR “Health Care Clinician*” OR “Health setting*” OR “Health Care Provider*” OR “nurse*” OR “doctor*” OR “primary care setting*” OR “antenatal service*” OR “mental health service*”].

### 2.2 Inclusion/Exclusion criteria

As the decision regarding which study designs to include in the review should be dictated by the review question (Nutbeam & Harris, 2004; Petticrew & Roberts, 2003), we included only qualitative studies as these would fully capture the victims' experiences and views of disclosing to the healthcare service. We also considered it important to synthesise qualitative evidence as this has been found to make a positive contribution to the knowledge available to international organisations, such as the World Health Organisation, when developing recommendations on public health topics (Metin Gülmezoglu et al., 2013).

Studies were further included if the study sample consisted of adult (16 years or older) domestic violence victims, who had experienced partner abuse. Thus, studies including victims of violence from other family members than the partner were excluded. Furthermore, the study should describe victims' experiences of disclosure/interactions within healthcare services (i.e., with professionals with health‐related qualifications, e.g., doctors, nurses, midwives). To safeguard study quality, readability and interpretability, we only included papers that were published in peer‐reviewed English language scientific journals. To capture most recent developments on barriers and facilitators of disclosure, we included papers published between 1996 and January 2018. Lastly, low quality studies (as determined by an adapted Critical Appraisal Skills Programme Checklist, CASP – see Section 3.4) were excluded.

### 2.3 Study selection

**FIGURE 1:**
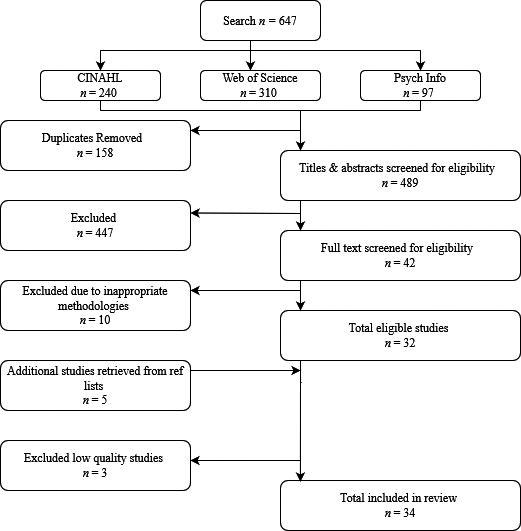
PRISMA flow diagram: A schematic view of study identification. PRISMA, Preferred Reporting Items for Systematic Reviews and Meta‐Analyses

All references were exported to Endnote Web. Our searches identified 647 papers, which was reduced to 489 after removal of duplicates. Titles and abstracts, as well as full text articles, were screened independently against inclusion and exclusion criteria by two reviewers. Disagreements were resolved through discussion until consensus was reached. Forty‐two papers underwent full text review, of which 32 studies met inclusion criteria. Additionally, five articles were identified by screening the reference lists of included studies. Quality assessment led to the exclusion of three low quality papers, reducing the total number of studies to 34. For a PRISMA flowchart see Figure 1.

### 2.4 Quality appraisal

The quality assessment was conducted with an adapted version of the CASP checklist for qualitative studies (Critical Appraisal Skills Programme, 2017). The quality of the studies was assessed on the domains research design, sampling and selection bias, attrition bias, performance bias, data collection, data analysis, and ethical issues (see Appendix S2 for the adapted checklist). Fourteen questions were answered to assess study quality in these domains and were answered positively with yes (+), negatively with no (−), or with not reported (N.R). A total score, number of unclear items, and a quality assessment score was given for each included article. The quality was rated on a rating scale from A – best (no risk of bias or only risk of bias in one of the assessed domains), B – good (risk of bias in two domains), C – sufficient (risk of bias in three domains), to D – insufficient (risk of bias in four or more domains). If the quality assessment score was D the study was excluded (see Appendix S3 for the methodological quality of included studies). To ensure reliability and uniformity of quality assessment, it was conducted independently by two evaluators. Any discrepancies in agreement were discussed and resolved and consensus was reached on the final quality assessment ratings by both reviewers.

### 2.5 Data extraction

Data were extracted on sample characteristics (e.g., age, gender), recruitment strategy and location, qualitative research methods and analysis, and findings of relevance to our research questions (barriers and facilitators, advice, and outcomes of disclosure), independently by two researchers. Differences in extracted information were discussed until consensus was reached.

## 3 RESULTS

### 3.1 Study characteristics

For a summary of findings from the included studies see Table 1. Seven hundred and eighty female victims and two male victims of domestic abuse were included across all studies, with an age range from 18 to 90 years old. The participants were recruited through domestic violence and women's services (12), maternity/antenatal clinics (6), print media, i.e., advertisements, articles in papers, public campaigns, and public safety announcements (5), emergency departments/hospitals (3), general practitioner's offices (3), a combination of recruitment strategies (2) and through mental health services (1). The majority of samples were recruited through purposeful sampling (25). The remaining nine studies reported using convenience sampling. Thirty studies were conducted in western countries, i.e., the United States of America (16), Australia (6), the United Kingdom (5), the Netherlands (1), Canada (1) and Scotland (1). Two studies were conducted in Asia (Nepal and Malaysia), one study was conducted in the Middle East (Jordan), and another study was conducted in South‐America (Mexico). Data were collected through interviews (28) and focus groups (6). Methods for analysis encompassed a variety of techniques, yet predominantly thematic analysis (14), grounded theory analysis (5) or content analysis (5).

**TABLE 1:** Study characteristics and main findings

Author, year and location	Participants & recruitment strategy & location	Data collection & analysis strategy	Findings	Relevance to research questions	Quality score
Bacchus et al. (2003) United Kingdom	16 females, 18 – 36 years Purposeful sampling Maternity clinic	Interviews Content analysis	*Barriers:* Feelings of fear and shame. No information about domestic violence in GP offices. Short appointments. Lack of continuity of care with HCPs. Lack of privacy during appointments with HCP. Negative attitudes about abuse of the HCP.	Q1	A
Bates et al. (2001) Australia	65 females, age range between 30 ‐ 60+ years Purposeful sampling Women's support services and refuges	Focus groups Thematic analysis	*Barriers:* Lack of privacy. Time constraints. Fearing children will be removed. Feelings of shame, guilt and powerlessness. Negative attitude of HCP. Lack of continuity of care with HCPs. *Facilitators:* Posters and pamphlets in waiting room. A safe and private place. Women preferred a female as a HCP *Advice:* Promote services available for DV victims, e.g., by providing written information. Have a specific domestic violence service provider.	Q1 and 2	A
Battaglia et al. (2003) USA	27 females, age range between 18 – 56 years Purposeful sampling Community‐based IPV organizations	Interviews Grounded theory method analysis	*Facilitators:* Communication, i.e., the HCP was willing to openly discuss abuse. Professional competency, i.e., the HCP was capable to talk about abuse. HCP was consistently accessible, respected confidentiality, and shared decision‐making. HCPs caring and not judging. Emotional equality, i.e., the HCP sharing personal information and feelings.	Q1	B
Bradbury‐Jones et al. (2011) Scotland	17 females, ages not given Purposeful sampling IPV support service	Interviews Thematic analysis	*Barriers:* Feelings of blame and low self‐esteem made the women less likely to use healthcare services. *Facilitators:* Establishment of trust, a positive relationship with the HCP, and not feeling judged.	Q1	A
Chang, Cluss, et al. (2005) USA	21 females, age range between 22 – 62 years Purposeful sampling Fliers and posters and directly from health clinicians	Interviews Grounded theory analysis	*Advice:* Make available posters/flyers/brochures with IPV support services and hotline numbers in waiting rooms and women's bathrooms. Provide information on legal help, i.e., custody children, divorce, and protection. Provide counselling regarding safety measures and mental health.	Q2	A
Chang, Decker, et al. (2005) USA	41 females, age range between 22 – 41 years Purposeful sampling IPV support group services	Focus groups Thematic analysis	*Advice:* Provide safety, support and information to access resources for DV victims regardless of disclosure.	Q2	B
Damra et al. (2015) Jordan	25 pregnant females, age range between 20 – 42 years Purposeful Sampling Maternity clinics	Interviews Thematic analysis	*Barriers:* Low perception of HCPs capabilities. Poor service sustainability. Lack of privacy, continuity of care, and time constraints. Perceived negative consequences of disclosure *Advice:* Directly asking about DV. The gender of the HCP, i.e., participants felt it was easier to talk to a female. *Outcomes:* Victims faced negative attitudes from the HCP, they were blamed and asked what it was that they did to make their husbands angry.	Q1, 2 and 3	B
Dienemann et al. (2005) USA	26 females, age range 21 – 65 years Purposeful sampling Hospitals and community domestic violence service	Focus groups Thematic analysis	*Advice:* HCPs should not tell a woman what to do, they should listen and only give suggestions. When screening HCPs should not only listen to the answers but also look at the non‐verbal cues. Make resources visible in the office to help physicians know how to respond to abuse	Q2	A
Gerbert et al. (1999) USA	25 females, age range 18 – 50 + years Purposeful sampling Print media (e.g., advertisements in newspapers and shelters)	Interviews Grounded theory analysis	*Barriers:* Denial of their situation. Lack of trust that HCP is capable of handling domestic abuse. Fear of losing children. Fear that confidentiality will be broken *Outcomes:* Over half of the participants described how their disclosure lead to a turning point in their life. For some of the women it made them realize the severity of their situation; in other cases it made them realize that they could leave their abusive relationship.	Q1 and 3	A
Gerbert et al. (1997) USA	31 females, age range between 21 – 59 years Convenience sampling Random digit dialing of households and by a publicity recruitment campaign.	Interviews Systems model analysis	*Barriers:* Fear of retaliation. Fear that children would be taken away. Feelings of shame, humiliation and embarrassment. Negative attitudes of the HCP. HCPs being too busy and the emergency room not being the appropriate place to discuss such topics. *Outcomes:* Seven of the women reported positive outcomes, they felt that the HCP was helpful and listened to their needs. The HCP did not pressure them to leave their abuser, but gave them support and information. Nine women reported negative experiences, they felt judged by the HCP. Other women felt ignored or stigmatized and some women were even made fun of.	Q1 and 3	C
Hathaway et al. (2002) USA	49 females, age range between 21 – 81 years Convenience sampling Hospital Based DV program	Interviews Content analysis	*Barriers:* Lack of time. Fear of their abuser and concerns about what will happen to their children. Feelings of shame and embarrassment. Not being aware of help that can be provided. *Facilitators:* The perceived knowledge or understanding the HCP has on the topic of abuse. Feeling like the HCP would keep their disclosure confidential. A good patient‐provider relationship; a caring, compassionate and open attitude of the HCP. Directly being asked about DV. Availability of posters and pamphlets with information about DV support. *Advice:* HCPs should not be judgemental and believe the victim. HCPs should not pressure women into leaving. Pay attention to safety and ensure that there is documentation of the abuse in case the patient wants to take legal action. *Outcomes:* Most of the women were referred to DV support services or mental health services after they disclosed. This made quite a few of the women feel supported.	Q1, 2 and 3	B
Hegarty and Taft (2001) Australia	20 females, no age range given, majority < 40 years. Purposive sampling Domestic violence service	Interviews Phenomenological analysis	*Barriers:* Feeling like they could deal with the abuse themselves, or that is was not severe enough to disclose. Feelings of shame, self‐blame, responsibility for family cohesion and that abuse was ‘normal’. Fear of their abuser and of being judged. *Facilitators:* HCPs were sympathetic, listened to their patient's problems, ensured confidentiality, and had the perceived potential to help.	Q1	B
Keeling and Fisher (2015) United Kingdom	15 females, age between 21 – 54 years Purposeful sampling Domestic violence support services	Interviews Thematic analysis	*Advice:* HCPs should be trained on how to react and deal with a DV disclosure. It is important that HCPs validate and support women who disclose DV to them. HCP should try not to dismiss disclosures as it could mirror perpetrators behaviours of minimization. *Outcomes:* Women who had positive responses (i.e., appropriate support and advice offered) after disclosure were able to leave violent relationships more often than women who had negative responses (i.e., whose disclosures were dismissed) when disclosing.	Q2 and 3	A
Kelly (2006) USA	17 self‐identified Latina women, age between 19 – 53 years Purposeful sampling Domestic violence service	Interviews Thematic analysis	*Barriers:* Fear of consequences for the children (either them living without a father or the children being taken away). Not being aware of their rights (either because of language barriers or because of avoidance of official agencies). Threats made by the abuser that if the abuse is disclosed they will kill them or their children. Lack of trust in the relationship with the HCP. *Advice:* Ask directly about DV. Put up posters and flyers in the office to make victims aware of their options.	Q1 and 2	A
Liebschutz et al. (2008) USA	27 females, age range between 18 – 56 years Purposeful sampling Domestic violence service	Interviews Grounded theory analysis	*Outcomes:* Fear and avoidance of seeking out healthcare after unhelpful/negative disclosure. Some of the women felt endangered after their disclosure. However, there was no actual increase in violence.	Q3	B
Lutenbacher et al. (2003) USA	24 females, age range between 21 – 51 years Convenience sampling Print media (advertisements)	Interviews and focus groups Systematic data analysis	*Barriers:* Not being asked directly about DV. Negative attitudes of the HCP (i.e., being ignored and not listened to). The abusers being present when the victims seek healthcare.	Q1	A
Lutz (2005) USA	12 females, age range between 18 ‐ 43 years Convenience sampling Prenatal clinics	Interviews Dimensional analysis	*Barriers:* Feelings of shame and fear. Fear of being negatively evaluated by their social environment. Fear of children being taken away.	Q1	A
McCauley et al. (1998) USA	21 females, age 18+ Purposeful sampling	Focus groups Thematic analysis	*Barriers:* Feelings of shame; denial that abuse was occurring; fear of a negative reaction of friends, family, or the HCP after disclosure; fear of the consequences to children; not feeling ready to change the relationship with the abuser; and fear of the abuser's reaction to disclosure. Lack of privacy or confidentiality and partner preventing access to medical care. Negative attitudes of the HCP. *Advice:* Women would be more inclined to discuss their abuse if they felt that the HCP was caring, easy to talk to, protective or offered a follow‐up.	Q1 and 2	B
Narula et al. (2012) Canada	10 females, age range between 40 – 73 years Convenience sampling General practitioners office	Interviews Content analysis	*Barriers:* Lack of insight into their situation, not all women felt they were being abused (i.e., in case of emotional abuse). Fear of for being judged or not believed or fear of threats made by the abuser. Feelings of shame and embarrassment. They felt like abuse is not a topic you discuss with your physician (e.g., rather discuss sleep disturbance). Lack of continuity of care and time constraints. *Advice:* The women advised the HCPs to listen to their needs, follow‐up after disclosure, offer validation, and provide support.	Q1 and 2	C
Nicolaidis, Gregg, Galian, McFarland, Curry and Gerrity (2008) USA	23 females, mean age 51.9 years Purposeful sampling General medicine clinic	Focus groups Thematic analysis	*Barriers:* Trust, i.e., if women felt that they could not trust their HCP then they were less likely to disclose. Fear that disclosing will lead to the HCP thinking that their physical symptoms are all in their head. *Advice:* Respect the patient and their understanding of their symptoms, their intelligence, strength and the complexity of their situation. Be honest and consistent.	Q1 and 2	B
Othman, Goddard and Piterman (2014) Malaysia	10 females, age range between 27 – 44 years Purposeful sampling Women's domestic violence shelter	Interviews Grounded theory analysis	*Barriers:* Victims felt that domestic violence is a private matter that should be resolved between the couple. They felt that DV is not something you should discuss with outsiders. Talking about their abuse was seen as embarrassing. Some of the women felt DV was their own fault viewed some abuse as normal. Fear of the abuser. Being financially dependent on the abuser. Not knowing what options and support are available. Fear of mandatory reporting or being forced to make a police report when they were not ready to leave yet.	Q1	C
Peckover (2003) UK	16 females, age not given Convenience sampling Domestic violence support services	Interviews Thematic analysis	*Barriers:* Fear that confidentiality will be broken and that they will lose their children. Since domestic violence is not viewed as a health topic, not all women felt it could be discussed with their HCP. No practical support or protection after disclosing. Language barrier prevented a victim from disclosing (need for interpreters). *Facilitators:* A good relationship with the HCP and privacy. The women who disclosed all did so during a home visit.	Q1	C
Reisenhofer and Seibold (2013) Australia	7 females, age range between 25 – 45 years Convenience sampling Emergency department and primary healthcare	Interviews Situational analysis	*Barriers:* Lack of privacy, i.e., the abuser is with them at the hospital. Previous negative reaction of HCP to disclosure will make it less likely they will disclose to other HCPs about their abuse. Feelings of fear, blame, and shame. Negative attitudes of the HCP. Not seeing their relationship as an abusive one or thinking that abuse is normal.	Q1	C
Rishal et al. (2016) Nepal	12 females, age range between 22 – 45 years Purposeful sampling Domestic violence and women's services	Interviews Graneheim and Lundman content analysis	*Barriers:* Fear of negative reactions from their friends and family, of being re‐victimized, and of being left by the abuser during pregnancy. No trusting relationship with the HCP, i.e., felt that they would be judged and that HCP would have no empathy. *Advice:* Ensure privacy and confidentiality. Make victims aware of resources for DV victims. The HCP should enquire about DV in a compassionate and empathetic way, without judgement	Q1 and 2	A
Rodriguez et al. (1996) USA	51 females, age range between 22 – 60 years Purposeful sampling Domestic violence services	Focus groups Thematic analysis	*Barriers:* Fear of abuse escalating. Low self‐esteem, shame and embarrassment. Sense of family responsibility, i.e., wanting to keep family together. Fear of being a single parent and being financially dependent on the abuser. *Facilitator:* A good patient–HCP relationship. *Advice:* Create a safe and supportive environment though compassion and understanding. Ask directly about abuse. Provide referrals and continued support.	Q1 and 2	A
Rose et al. (2011) UK	16 females and 2 males, age range 19 – 59 years Purposive sampling Community mental health services	Interviews Thematic analysis	*Barriers:* Fear, including fear of social services; fear that they will not be believed; fear that disclosure will lead to more violence; fear to disrupt the family and fear of the consequences for one's immigration status. Self‐blame, shame and embarrassment. Social isolation and lack of a support network. *Facilitators:* A good and supportive relationship with the HCP.	Q1	B
Salmon et al. (2015) United Kingdom	7 females, age range between 24 – 38 years Purposeful sampling Community antenatal clinic	Interviews Constant comparative analysis	*Barriers:* Embarrassment. Fear of not being believed. Fear of the consequences of disclosing, especially losing the children. Receiving vague questions instead of directly being asked about DV.	Q1	B
Spangaro, Herring, et al. (2016) Australia	12 pregnant aboriginal females, age range between 20 – 36 years Purposeful sampling Maternal hospital	Interviews Qualitative configurative analysis	*Barriers:* Fear of consequences, i.e., abuser finding out, child being taken away. Lack of care, i.e., the HCP does not come across as caring, empathetic and trustworthy. *Facilitators:* Direct asking, caring, safety from institutional control, the abuser, shame, and culture Advice Ask directly about abuse	Q1 and Q2	A
Spangaro, Koziol‐McLain, et al. (2016) Australia	32 females, age range between 17 – 41 years Convenience sampling Antenatal clinic	Interviews Qualitative configurative analysis	*Barriers:* Non‐caring HCP, i.e., closed body language; HCP reading off the computer screen; impersonal attitude; not being able to ask questions; and unexplained departures from the room. Shame and fear about the situation. Not wanting to relive the trauma. Fear of institutional control, such as social services taking the children.	Q1	A
Spangaro et al. (2011) Australia	20 females, age range between 17 – 50 years Volunteer sampling Antenatal, mental health and substance abuse services	Interviews Inductive analysis	*Barriers:* Fear for safety from abuser, feelings of shame and embarrassment, and fear of institutional control. *Facilitators:* Direct asking, perceived trustworthiness of HCP, and choice, i.e., feeling like they get to decide how much and what to say. *Outcomes:* For several of the women being asked about abuse made them rethink their relationship and realize that they were abused. For some it made them realize that they were not at fault, for others it made them aware of support services.	Q1 and 3	A
Wallin Lundell et al. (2018) Mexico	7 females, age range between 21 – 49 Purposeful sampling Hospital	Interviews Content analysis	*Barriers:* Feelings of guilt, such as thinking one is to blame for the abuse, which at times gets reinforced by the attitude and actions of HCPs. Lack of interest by the HCP, feeling like they were not taken seriously. Time constraints, i.e., most of the women felt that the HCP did not have time for them to tell their whole story. Not feeling secure or able to trust the HCP. Lack of confidentiality.	Q1	C
Wong et al. (2008) The Netherlands	36 females, age range between 17 – 56 + years Purposeful sampling Family doctor	Interviews Thematic analysis	*Facilitators:* Empathetic and empowering approach; HCP initiating the subject and asking direct questions, offering support. *Outcomes:* Most participants felt relieved, calm and satisfied after disclosing. Four weeks after disclosing 20 participants felt optimistic (perceiving a change in their ability to handle the situation). They were taking steps and considering the benefits to their children. Thirteen of the participants felt no improvement in their situation, they mainly emphasized their fear and inability to do anything to improve their situation.	Q1 and 3	A
Yam (2000) USA	5 females, age range from 22 – 36 years Purposeful sampling Emergency department	Interviews Thematic analysis	*Barriers:* Feelings of shame, embarrassment, fear for partner and concern for their children. Feeling that the HCP does not understand their situation, does not take it seriously or in some cases that the HCP blames them for their situation. Negative attitudes of and relationship with the HCP. The HCP seems uncaring, unsympathetic, rushed or unwilling to discuss abuse. Lack of resources/support system. *Advice:* HCPs should express compassion, ensure advocates are available, make the women feel safe, provide them with options, listen to them and discuss disclosure privately. Follow‐up on the patient. Have a trained counsellor and advocate for victims available.	Q1 and 2	B
Zink et al. (2004) USA	38 females, age range between 55 −90 years Convenience sampling Public advertisement	Interviews Immersion crystallization techniques	*Barriers:* Feelings of shame, commitment to their abuser and not realizing they were in an abusive relationship. Victims also felt that their upbringing had taught them to stay with their husband and that they should not challenge him. Lack of time and privacy. *Advice:* Listen to needs of patients and having an empathic response. Not judging the women for not leaving or pressuring them into leaving. Raising the subject of DV, so women know they can talk about it *Outcomes:* Several of the women had negative experiences after disclosing. Providers ignored their disclosure or were uncomfortable with discussing it. This caused these women not receiving the validation or assistance they were seeking. There was also a lack of empathy because the women would not leave their husbands. In contrast over half the women felt positive about their disclosure. Their HCPs were helpful, listened and gave referrals for support, they validated the women and in some cases, this lead to the women leaving their abuser	Q1, 2 and 3	B

Abbreviations: advice; DV, domestic violence; facilitators and barriers; GP, general practitioner; HCP, healthcare professional; IPV, intimate partner violence; outcomes; Q1, question 1; Q2, question 2; Q3, question 3.

### 3.2 Study quality

Three of the studies were of insufficient quality and thus excluded. Sixteen of the studies included in our review were rated as A – best (no risk of bias or only risk of bias in one of the assessed domains), 12 were rated as B – good (risk of bias in two domains) and six were rated as C – sufficient (risk of bias in three domains). The quality criteria that were most often judged as low was that of attrition bias (in all 34 included studies) and performance bias (in 16 included studies). Study quality was not explicitly considered when interpreting the findings, as the majority of studies were of good quality and variability in study quality was limited.

### 3.3 Main findings

Themes for each article were identified and grouped under three main headings: barriers and facilitators of disclosure, advice to improve disclosure and outcomes of disclosure.

#### 3.3.1 Barriers and facilitators of disclosure

Of the 34 articles included in this review, 28 discussed barriers and/or facilitators for disclosure. Most of the articles (27) discussed barriers, and 11 (additionally) discussed facilitators.

##### Barriers to disclosure

One of the barriers that the victims often mentioned was a fear of the consequences that disclosing domestic abuse could have. The most feared consequence of disclosing was the fear that their children would be taken away (Bates et al., 2001; Gerbert et al., 1997, 1999; Hathaway et al., 2002; Kelley, 2006; Lutz, 2005; McCauley et al., 1998; Peckover, 2003; Salmon et al., 2015; Spangaro, Herring, et al., 2016; Spangaro, Koziol‐McLain, et al., 2016).

Secondly, victims feared being judged/ negatively evaluated by either the HCP or their environment, i.e., their family, friends, neighbours, acquaintances (Damra et al., 2015; Hegarty & Taft, 2001; Lutz, 2005; McCauley et al., 1998; Narula et al., 2012; Othman et al., 2014; Rishal et al., 2016) and some victims feared that they would not be believed (Narula et al., 2012; Rose et al., 2011; Salmon et al., 2015).

Thirdly, victims communicated that fear of their abuser further prevented them from disclosing. For instance, they were fearful of what would happen if their abuser found out about their disclosure and they were worried about whether the healthcare service would be able to protect them, especially as in many cases their abuser made threats prior to them disclosing (Hathaway et al., 2002; Hegarty & Taft, 2001; Kelley, 2006; Lutenbacher et al., 2003; McCauley et al., 1998; Narula et al., 2012; Othman et al., 2014; Peckover, 2003; Rodriguez et al., 1996; Rose et al., 2011; Spangaro et al., 2011; Yam, 2000). Relatedly, three studies found that victims feared that confidentiality would be broken by the health service personnel (Gerbert et al., 1999; Peckover, 2003; Wallin et al., 2003) and they were also concerned about the lack of privacy at the healthcare service (Bacchus et al., 2003; Bates et al., 2001; Damra et al., 2015; McCauley et al., 1998; Reisenhofer & Siebold, 2013; Zink et al., 2004).

A lack of a positive relationship with the HCP was also viewed as a barrier to disclosure (Bacchus et al., 2003; Bates et al., 2001; Damra et al., 2015; Hathaway et al., 2002; Kelly, 2006; Narula et al., 2012; Nicolaidis et al., 2008; Rishal et al., 2016; Spangaro, Herring, et al., 2016; Spangaro, Koziol‐McLain, et al., 2016; Wallin et al., 2018; Yam, 2000). This included not trusting in the HCP, lack of continuity in the relationship or limited time with the HCP, and not expecting that the HCP would be empathetic.

Furthermore, the HCP having a negative attitude towards them or their disclosure, for example, by being unsympathetic, disinterested, not maintaining eye contact or not listening were also viewed as barriers to disclosure (Bacchus et al., 2003; Bates et al., 2001; Gerbert et al., 1997; Lutenbacher et al., 2003; McCauley et al., 1998; Reisenhofer & Seibold, 2013; Spangaro, Herring, et al., 2016; Spangaro, Koziol‐McLain, et al., 2016; Wallin et al., 2018; Yam, 2000). Victims perceptions of whether the HCP could handle their disclosure also acted as a barrier, specifically perceiving the HCP to have low capability to help them prevented victims from disclosing (Damra et al., 2015; Gerbert et al., 1999; Lutenbacher et al., 2003).

Additionally, personal barriers such as a low self‐esteem, feelings of shame, embarrassment, guilt and powerlessness also prevented victims from disclosing (Bacchus et al., 2003; Bates et al., 2001; Bradbury‐Jones et al., 2011; Gerbert et al., 1997; Hathaway et al., 2002; Hegarty & Taft, 2001; Lutz, 2005; McCauley et al., 1998; Narula et al., 2012; Reisenhofer & Seibold, 2013; Rodriguez et al., 1996; Rose et al., 2011; Salmon et al., 2015; Spangaro et al., 2011; Spangaro, Koziol‐McLain, et al., 2016; Wallin et al., 2018; Yam, 2000; Zink et al., 2004). Furthermore, victims chose not to disclose because they were at the time unaware that they were experiencing abuse (Narula et al., 2012; Zink et al., 2004). Some of the victims reported thinking that the abuse was normal and something that you just tolerate in a relationship (Hegarty & Taft, 2001; Othman et al., 2014; Reisenhofer & Seibold, 2013; Zink et al., 2004). Other reasons victims were reluctant to disclose was being in denial (Gerbert et al., 1999), not being ready to leave their abuser (McCauley et al., 1998; Othman et al., 2014), being financially dependent (Othman et al., 2014; Rodriguez et al., 1996), lacking social support (Rose et al., 2011; Yam, 2000) and trying to avoid reliving the trauma by not speaking about the abuse (Spangaro, Koziol‐McLain, et al., 2016).

A further barrier was the fact that many victims thought that healthcare services were not the appropriate place to discuss abuse, as they perceived that it was not a health issue that you discuss with a HCP (Gerbert et al., 1997; Narula et al., 2012; Othman et al., 2014; Peckover, 2003). Additionally, victims were also not always aware of their rights in terms of what choices or support they could gain by telling the HCP about abuse (Hathaway et al., 2002; Kelly, 2006; Othman et al., 2014).

##### Facilitators to disclosure

The 11 studies focused on facilitators for disclosure generally found that a positive and trusting relationship with the HCP was important for victims to enable disclosure (Bates et al., 2001; Battaglia et al., 2003; Bradbury‐Jones et al., 2011; Hathaway et al., 2002; Hegarty & Taft, 2001; Peckover, 2003; Rodriguez et al., 1996; Rose et al., 2011; Spangaro, Herring, et al., 2016; Spangaro et al., 2011; Wong et al., 2008).

Other studies found that HCPs directly asking about abuse facilitated victims' disclosures (Hathaway et al., 2002; Spangaro, Herring, et al., 2016; Spangaro et al., 2011; Wong et al., 2008). Additionally, victims stated that when they felt that the healthcare setting was safe and they were convinced that their disclosure would be kept confidential, they were more likely to disclose (Bates et al., 2001; Battaglia et al., 2003; Hathaway et al., 2002; Hegarty & Taft, 2001; Peckover, 2003; Spangaro, Herring, et al., 2016; Spangaro et al., 2011).

Additional factors facilitating victims' disclosures were perceiving the HCP as knowledgeable and capable to handle the abuse (Battaglia et al., 2003; Hathaway et al., 2002), and the gender of the HCP; a woman facilitated disclosure among female victims (Bates et al., 2001). Victims further noted that feeling that they had a choice over what to disclose and how much, helped them to disclose (Spangaro, Herring, et al., 2016; Spangaro et al., 2011). Finally, having leaflets and posters on domestic violence visible and available in waiting rooms further aided victims' disclosures (Bates et al., 2001).

#### 3.3.2 Advice to improve disclosure

Of the 34 studies included in the review, 16 contained advice from victims on how the healthcare service may improve responding to victims' disclosures.

A commonly given advice was to make victims aware of the resources and options available to disclose in the healthcare service. This could either be by making posters and pamphlets visible and available in the waiting room or by talking about resources during the appointment (Bates et al., 2001; Chang, Cluss, et al., 2005; Chang, Decker, et al., 2005; Dienemann et al., 2005; Kelly, 2006; Rishal et al., 2016). Another oft‐mentioned advice was for HCPs to directly ask their patients about domestic abuse (Damra et al., 2015; Kelly, 2006; Rodriguez et al., 1996; Spangaro, Herring, et al., 2016).

It was further advised that HCPs put in an effort to make the environment safe, private and confidential for disclosure (Chang, Decker, et al., 2005; Rishal et al., 2016; Rodriguez et al., 1996; Yam, 2000). HCPs were also advised to be aware of how their attitude could influence the choice of a victim to disclose. Victims advised that a caring, non‐judgemental and supportive attitude is what would facilitate disclosure and they stressed the importance of respecting the victim and showing compassion (Keeling & Fisher, 2015; McCauley et al., 1998; Nicoladis et al., 2008; Rishal et al., 2016; Rodriguez et al., 1996; Yam, 2000; Zink et al., 2004).

Female victims also communicated finding it easier to talk about abuse to another woman (Damra et al., 2015) and so they advised that victims should be provided with an option to meet with a female HCP. Furthermore, it was advised that HCPs provide referrals and continued support (Narula et al., 2012; Rodriguez et al., 1996; Yam, 2000) and that they are specially trained to deal with domestic violence victims disclosures (Bates et al., 2001). It was also advised to make a mental health counsellor and a legal counsellor available (Chang, Cluss, et al., 2005; Yam, 2000). Lastly, victims recommended not to pressure patients into leaving their abuser (Hathaway et al., 2002; Zink et al., 2004).

#### 3.3.3 Outcomes of disclosure

Nine of the 34 included articles discussed what victims experienced after their disclosure of domestic abuse to a HCP. The studies discussed both positive and negative outcomes of disclosure of domestic violence.

Positive outcomes of disclosure included victims feeling disclosure was a turning point (Gerbert et al., 1999), feeling validated (Gerbert et al., 1997; Hathaway et al., 2002; Zink et al., 2004), feeling optimistic about becoming free from violence (Wong et al., 2008), rethinking one's relationship, reductions in feelings of self‐blame, becoming aware of available support (Keeling & Fisher, 2015; Spangaro et al., 2011), and, ultimately, direct changes through filing of a police report and leaving one's abuser (Liebschutz et al., 2008). The latter steps appear particularly likely to be undertaken when initial responses to disclosure were a positive experience.

However, some of the studies from our review also reported negative outcomes of disclosure. For example, victims noted no change or improvement in their situations after disclosing (Damra et al., 2015; Wong et al., 2008) and instead feeling blamed, stigmatized or ignored by HCPs (Damra et al., 2015; Gerbert et al., 1997; Zink et al., 2004). After disclosing, some victims also reported feeling helpless and fearful (Wong et al., 2008) noting that they felt even more endangered after disclosing abuse (Liebschutz et al., 2008).

## 4 DISCUSSION

The aim of this systematic review was to synthesize qualitative research on the barriers and facilitators of disclosure that victims of domestic violence experience in healthcare settings. We also synthesized the advice that victims provide on the ways in which healthcare services can increase victims' disclosures of domestic abuse, and what outcomes they perceived after disclosing domestic abuse to a HCP.

Generally speaking, this review has demonstrated a continued scientific interest in domestic violence victims' experiences of disclosure in healthcare settings. Over the past 12 years, more than twice as many qualitative papers were written about this topic than in the 10 years preceding it. A majority of studies focus on barriers to disclosure, but facilitators of disclosure, victims' advice to HCPs, and outcomes of disclosure are increasingly investigated as well.

Barriers to disclosure within this review can be divided into two parts. Firstly, there are barriers related to opinions and feelings of the victims. These barriers consisted of victims' fear of their children being taken away by child protection services, their abuser, and being negatively judged by the HCP and/or their social environment. Victims also noted feelings of guilt, shame, embarrassment and not realizing that what they were experiencing was abuse, further acted as barriers to them disclosing, along with perceptions that the HCP was not competent or capable to handle their domestic abuse disclosure (Bacchus et al., 2003; Bates et al., 2001; Bradbury‐Jones et al., 2011; Gerbert et al., 1997; Gerbert et al., 1999; Hathaway et al., 2002; Hegarty & Taft, 2001; Kelly, 2006; Lutz, 2005; McCauley et al., 1998; Narula et al., 2012; Nicolaidis et al., 2008; Othman et al., 2014; Reisenhofer & Seibold, 2013; Rishal et al., 2016; Rodriguez et al., 1996; Rose et al., 2011; Salmon et al., 2015; Spangaro et al., 2011; Spangaro, Herring, et al., 2016, Spangaro, Koziol‐McLain, et al., 2016; Wallin et al., 2018; Yam, 2000; Zink et al., 2004).

Secondly, victims also experienced institutional barriers which prevented them from disclosing. These included the inability to form a trusting relationship with the HCP, lack of continuity in care, and limited time with the HCP (Bacchus et al., 2003; Bates et al., 2001; Damra et al., 2015; Gerbert et al., 1997; Hathaway et al., 2002; Lutenbacher et al., 2003; Narula et al., 2012; Peckover, 2003; Rishal et al., 2016; Spangaro, Herring, et al., 2016; Spangaro et al., 2011; Yam, 2000; Zink et al., 2004). Additionally, victims encountered a further institutional barrier of lack of privacy, which further prevented them from disclosing to HCPs (Bacchus et al., 2003; Bates et al., 2001; Damra et al., 2015; Reisenhof & Seibold, 2013; Zink et al., 2004).

Facilitators of disclosure included a trusting relationship with the HCP, directly being asked about domestic abuse, the availability of pamphlets and posters for domestic violence services in the waiting rooms, having privacy, and the option to see a female HCP for female victims (Bates et al., 2001; Battaglia et al., 2003; Bradbury‐Jones et al., 2011; Hathaway et al., 2002; Hegarty & Taft, 2001; Peckover, 2003; Rodriguez et al., 1996; Rose et al., 2011; Spangaro et al., 2011; Spangaro, Herring, et al., 2016; Wong et al., 2008).

When comparing present results against Robinson and Spilbury's (2008) review, two findings stand out. First, a substantial part of earlier findings on barriers and facilitators of disclosure have been replicated in more recent work. For example, a lack of a positive relationship with the HCP, the HCP having a negative attitude, the absence of privacy and fear of losing one's children have all been repeatedly identified as barriers preventing victims from disclosing domestic abuse. However, in recent years additional barriers were also identified. This included victims fearing not being believed by HCPs (Narula et al., 2012; Rose et al., 2011; Salmon et al., 2015), being concerned about re‐living the trauma when disclosing to a HCP (Spangaro, Koziol‐McLain, et al., 2016), having perception that the HCP is incapable to respond to disclosure (Damra et al., 2015), and a lack of eye contact with clients due to the use of computer screens (Spangaro, Koziol‐McLain, et al., 2016). Despite the development of national (e.g., National Institute for Health & Care Excellence [NICE], 2014) and international guidelines (Department of Health, 2000; World Health Organisation, 2013b) and repeated recommendations by researchers on how HCPs can facilitate disclosure of abuse (Feder et al., 2011; Nyame et al., 2013), our review illustrates both the variety of barriers to disclosure and the continuity of such barriers over time and potentially across cultures.

Similarly, identified facilitators show some consistency across time. For example, both Robinson and Spilsbury's (2008) review and our review found that a positive and trusting relationship with the HCP, safety, the HCP being knowledgeable and ensuring that leaflets and posters were available within the healthcare setting facilitated disclosure. However, our review also identified new facilitators of disclosure such as victims having autonomy over what to disclose and how much (Spangaro, Herring, et al., 2016; Spangaro et al., 2011) and them being questioned directly about abuse (Hathaway et al., 2002; Spangaro, Herring, et al., 2016; Spangaro et al., 2011; Wong et al., 2008). The findings from both our review therefore highlight that disclosure can be improved for victims. We recommended that HCPs be educated upon these ways with training, especially as training can help HCPs identify and respond better to victims of domestic abuse (Beynon et al., 2012).

Relatedly, when comparing results from our review with Robinson and Spilsbury's (2008) review, some consistency on advice provided by domestic violence victims to HCPs is apparent. While the prior review did not specifically set out to synthesize advice, it did show that when victims provide advice, this was in many respects similar as the advice identified in our review. For instance, many of the included studies in this review recommended that HCPs ensure that posters and pamphlets are made available (Bates et al., 2001; Chang, Cluss, et al., 2005; Chang, Decker, et al., 2005; Dienemann et al., 2005; Kelly, 2006; Rishal et al., 2016), that the environment is kept private and confidential to help victims to feel safe (Chang, Cluss, et al., 2005; Rishal et al., 2016; Yam, 2000; Zink et al., 2004), that victims disclosures should not be judged, that victims should not be pressured into leaving (Hathaway et al., 2002; Rishal et al., 2016; Zink et al., 2004), and that victims be provided with counselling and continued support after disclosing (Chang, Decker, et al., 2005; Narula et al., 2012; Rishal et al., 2016). However, our review also uniquely identified new advice such as that direct questioning should be carried out by HCPs (Damra et al., 2015; Kelly, 2006; Spangaro, Herring, et al., 2016). It should be noted though that screening has not always been supported in previous studies as it may negatively impact on women's mental health (Klevens et al., 2012). Other new advice included providing training to HCPs on how to react and deal with a domestic violence disclosure and avoiding minimizing victims' experiences of domestic violence when disclosing, as this could mirror perpetrators minimization of abuse (Keeling & Fisher, 2015).

With respect to the outcomes of disclosure, which was not covered by Robinson and Spilsbury (2008), a nuanced picture emerges. On the one hand, disclosure can help victims to feel validated and supported (Gerbert et al., 1997; Hathaway et al., 2002; Zink et al., 2004), which can be experienced as a turning point (Gerbert et al., 1999; Spangaro et al., 2011) resulting in victims deciding to leave their abuser (Liebschutz et al., 2008), or becoming aware of what support is available (Keeling & Fisher, 2015; Wong et al., 2008). Such findings are generally in line with quantitative research, which has for example demonstrated that approximately half of domestic violence victims can become free from violence within 6 weeks after disclosure if provided with appropriate care (Krasnoff & Moscati, 2002). However, on the other hand, potential negative consequences of disclosure should not be disregarded. For example, negative responses from HCPs can lead to victims feeling ignored, stigmatized (Gerbert et al., 1997; Zink et al., 2004), and blamed (Damra et al., 2015), resulting in them finding it more difficult to leave. Our review also highlighted how some victims could feel even more endangered (Liebschutz et al., 2008) and helpless to change their situations (Wong et al., 2008).

### 4.1 Implication for policy and practice

Based on the above, we conclude that training is important and should be provided to HCPs to help them to understand the complexities of the disclosure process and the variety of potential barriers and facilitators of disclosure that may be experienced. For example, research shows that HCPs would rather not ask about domestic violence because it makes them uncomfortable or afraid of offending their patients (Hamberger et al., 1998). However, several studies in our review showed that victims of domestic abuse would prefer being asked directly about abuse (e.g., Damra et al., 2015; Kelly, 2006). Thus, HCPs should be trained to help them understand what questions to ask under which circumstances. For example, HCPs could start off with indirect questions such as ‘Do you feel comfortable in your home’ or ‘do you feel in control of your life’ (Fulfer et al., 2007), and then gradually they could ask more direct questions such as, ‘Are you experiencing abuse emotional, physical or sexual?’ Training has been shown to be more effective if professionals are given the opportunity to observe and model good practice. For example, experiential and interactive training has been found to be effective in previous studies with HCPs (Haney et al., 2003; Zaher et al., 2014). Haney et al. (2003) demonstrated that HCPs confidence in asking about and responding to domestic violence markedly improved after completing an interactive training.

Additionally, healthcare services should take into account barriers unrelated to HCPs training and abilities. For instance, a lack of information about domestic violence, a lack of privacy or a lack of opportunity to disclose to a female HCP, all act as barriers to disclosure, yet could be targeted by implementing structural changes in healthcare facilities (Bacchus et al., 2003; Bates et al., 2001; McCauley et al., 1998; Reisenhofer & Seibold, 2013; Zink et al., 2004). For example, creating private areas for contact with female HCPs in Accident and Emergency hospitals or maternity clinics could directly reduce a barrier to disclosure. This might also help reduce the emotional barrier of feelings of shame and embarrassment. That is, if fewer people interact with a victim in a private environment, this could make her feel more comfortable to openly discuss her experiences.

Additionally, based on the findings from our review, we concluded that victims may find it difficult to disclose due to fears that their children will be removed. Victims' anxiety may be warranted as guidelines have highlighted that general practitioners do not always know what to do when presented with child safeguarding cases of domestic violence (General Medical Council, 2012). Thus, it is recommended that training is provided to HCP's and that they are reminded to be sensitive towards victims, making every effort not to blame them for difficulties they experience in protecting their children due to domestic violence (Lapierre, 2008; Radford & Hester, 2006). Instead HCP's should work closely with child protective services to ensure that victims are fully supported to be able to care and protect their children. Research by Mullender et al. (2002) also showed that children (aged 8–17) who were living in homes with domestic violence, wished to be treated as agentic and be involved in the decision‐making process on solutions to this problem, including whether they want to remain in their mother's care. These measures, combined with the provision of information on how child safeguarding is handled by healthcare professionals (e.g., in information brochures on domestic violence) could reduce victims fears about losing their children after disclosing.

Finally, after acknowledging victims' recommendations for what can improve disclosure, we also must not forget that disclosure does not always lead to positive consequences and may result in more harm if not responded to correctly (Liebschutz et al., 2008). Victims in one study advised that HCPs should be trained specifically on how to respond and deal with a disclosure (Keeling & Fisher, 2015). We need to ensure that we optimize the possibility of positive outcomes for victims when disclosing, especially since disclosure can lead to victims becoming free from violence if they are provided with appropriate care (Krasnoff & Moscati, 2002).

Appropriate care based on the qualitative findings from this review from the perspective of the victims would consist of the HCP responding in an empathic (e.g., ‘I am so sorry that this is happening to you’), validating (e.g., ‘you are correct what you are experiencing is abuse’), non‐judgemental (e.g., ‘You do not deserve this and none of this is your fault’) and non‐pressurizing way (e.g., ‘feel free to discuss with me when you feel ready’), within a safe and private environment (in a closed soundproof room without their partner present). Such a response from The HCP will allow the victim to (begin) establishing trust with the HCP and form a better relationship. To increase the odds of positive outcomes of disclosure, the HCP should have up to date information on where to refer the victim for appropriate support (e.g., women's shelters, legal advocacy), or care (e.g., counselling) so that the victim can be made aware of all their options. While not all victims will be at imminent risk of violence, the HCP should assess the victim's risk of violence, which would allow them to create a safety plan with the victim if needed, e.g., advising the victim to store money/documents etc so they can escape urgently if needed. HCPs should ideally also be trained to use tools, which could help them to assess the victim's risk, i.e., screening measures such as the woman abuse screening tool (Basile et al., 2007). Finally, after disclosure, the HCP should end their consultation by acknowledging the victim's courage to disclose abuse (‘I am very proud of you, it must not be easy to open up about your experiences’) and provide a follow‐up appointment if needed.

### 4.2 Limitations and future research

Multiple considerations should be taken into account when interpreting our findings. Some limitations relate to methodological choices. First, we only synthesised qualitative studies, as this would provide us with rich data, which would help us to understand victims' experiences and perspectives through their own voices. Due to the subjective nature of qualitative research various biases may have shaped results from our review. For example, social desirability may have influenced answers in both the interview or focus group studies, as participants may have adapted their answers due to wanting to be accepted or liked by the researchers or group members. A further limitation of this review was that it did not consider non‐English peer reviewed articles, or grey literature (dissertations, master theses), to safeguard interpretability of results. Future reviewers focusing on these topics could aim to be more comprehensive in their selection of manuscripts. A further limitation of our review was that we grouped findings on our main research questions together for all types of HCPs. While we are confident that the conclusions from our review are broadly applicable, some barriers and facilitators may be unique to specific settings or HCPs. For instance, unique barriers to victim disclosure exist within mental healthcare (Rose et al., 2011; Trevillion et al., 2016), such as concerns that mental illness is viewed as the main cause of abuse, rather than (other) personal or social factors (Du Mont & Forte, 2014).

Other limitations pertain to the acquired data. One of the limitations of this study is the lack of male participants in the included studies; only two male participants were included in our review. Relatedly, only 12% of victims included in our review were from non‐Western countries. Therefore, an important aim for future research is establishing if the present results generalize to male domestic violence victims, and victims from non‐western countries. The studies included in this review were deemed to be of sufficient to excellent quality, however, all of the studies had some level of attrition bias and selection/sampling biases were common. Future studies should aim to address these biases.

## 5 CONCLUSION

In summary, the present study strengthened the research base on disclosure of domestic violence in healthcare settings by providing a comprehensive, updated systematic review of qualitative research barriers and facilitators of disclosure, advice of victims on disclosure, and the potential outcomes of disclosure. Results were partly consistent with a prior review, but also demonstrated novel themes and issues relevant to disclosure in healthcare settings. The fact that barriers to disclosure persist despite the development of international guidelines and regulations for healthcare services suggests that improving HCP and healthcare service responsiveness to domestic violence remains an important goal for the future.

## CONFLICT OF INTEREST

The authors declare no conflicts of interest.

## Supporting information

Supplementary Material

Supplementary Material

## Data Availability

The data that support the findings of this study are available in Table 1 and the Supporting Information of this article.
