# Supplementary material for: Barriers and facilitators of disclosing domestic violence to the healthcare service: A systematic review of qualitative research
Source: Health Soc Care Community. 2021 Jan 13;29(3):612–30. doi: 10.1111/hsc.13282 (PMC8248429; doi:10.1111/hsc.13282)
Supplement: Supplementary file 1 — Supplementary Material [file HSC-29-612-s001.docx]

**Appendix A:** **Database, year searched, search terms and number of hits**

| **Database** | **Date searched** | **Search terms** | **Number of hits** |
| --- | --- | --- | --- |
| PsychINFO | January 2018 | "Domestic* violence*" OR "battered female*" OR "intimate partner violence” OR “partner abuse*” OR “domestic abuse*” OR “battered men*” OR “battered male**” OR “battered women*” OR “victim*” OR “spouse abuse*” OR “survivor*” OR “female survivor*” OR “male survivor*” OR “intimate partner violence survivor*” ) AND ( “Self-Disclosure” OR "Disclosure*" OR "help seeking" ) AND ( “Health care service*”OR “Health care professional*” OR “Health Care Clinician*” OR “Health setting*” OR “Health Care Provider*” OR “nurse*” OR “doctor*” OR “primary care setting*” OR “antenatal service*” OR “mental health service*” | 97 |
| CINAHL | January 2018 | ( "Domestic* violence*" OR "battered female*" OR "intimate partner violence” OR “partner abuse*” OR “domestic abuse*” OR “battered men*” OR “battered male**” OR “battered women*” OR “victim*” OR “spouse abuse*” OR “survivor*” OR “female survivor*” OR “male survivor*” OR “intimate partner violence survivor*” ) AND ( “Self-Disclosure” OR "Disclosure*" OR "help seeking" ) AND ( “Health care service*”OR “Health care professional*” OR “Health Care Clinician*” OR “Health setting*” OR “Health Care Provider*” OR “nurse*” OR “doctor*” OR “primary care setting*” OR “antenatal service*” OR “mental health service*” ) | *240* |
| WEB OF SCIENCE | January 2018 | TOPIC: ("Domestic* violence*" OR "battered female*" OR "intimate partner violence" OR "partner abuse*" OR "domestic abuse*" OR "battered men*" OR "battered male**" OR "battered women*" OR "victim*" OR "spouse abuse*" OR "survivor*" OR "female survivor*" OR "male survivor*" OR "intimate partner violence survivor*") AND TOPIC: ("Self-Disclosure" OR "Disclosure*" OR "help seeking") AND TOPIC: (“Health care service*”OR “Health care professional*” OR “Health Care Clinician*” OR “Health setting*” OR “Health Care Provider*” OR “nurse*” OR “doctor*” OR “primary care setting*” OR “antenatal service*” OR “mental health service*”) | *310* |

**Appendix B: Adapted quality assessment checklist**

**Qualitative studies**

**Screening question**

1. Were the aims and objectives of the study made clear enough (i.e., did the study aim to focus on victim’s experiences/perceptions of disclosure to the health service)? **Y/N/U comments**

**Appropriate research design**

1. Was the research design used appropriate to address the aims of the research? (i.e., does the research seeks to interpret or illuminate the actions and/or subjective experiences of research participants? Is qualitative research the right methodology for addressing the research goal? did the researchers explain and justify why they used particular methods) **Y/N/U comments**

**Sampling and selection bias**

1. Was an appropriate sample obtained, i.e., all victims of domestic violence and were details given as to how the sample was identified and recruited? **Y/N/U comments**
2. Did the study use a large enough sample size to reach data saturation (minimum 12 participants or if less the researchers took into consideration data saturation)? **Y/N/U comments**

**Attrition bias (withdrawal’s)**

1. Were there any participants who dropped out of the study? **Y/N/U comments**
2. Were victims who participated the same as those who did not? **Y/N/U comments**
3. Was there any attempt to deal with missing data? **Y/N/U comments**

**Performance bias (reflexivity)**

1. Did the researcher consider their role in gathering information from the participant (i.e., if the researcher was a health professional themselves did they consider any potential biases or influence they may have had on the participants response)? **Y/N/U comments**

**Data collection**

1. Was the data collected appropriately for a qualitative study, i.e., (transcribes, tape recordings, focus groups, interviews etc. and was sufficient detail given explaining why that type of data collection was used? **Y/N/U comments**

**Data analysis**

1. Was the data analysis sufficiently rigorous? (i.e., are coding and analytical strategies clearly described) Y/N/U comments
2. Is the data in depth and ‘rich’? **Y/N/U comments**
3. Did the study show attempts to establish reliability and validity of data analysis (i.e., was there more than one analyst providing consistency)? **Y/N/U comments**
4. Does the study include sufficient original data, i.e., (direct quotations from interview transcripts/ data tables etc.) which can justify the interpretations or conclusions which were drawn? **Y/N/U comments**

**Ethical issues**

1. Have ethical issues been taken into consideration, i.e., (was the study approved by a research ethics committee, was informed consent obtained and were details given of how the researcher handled the effects of the study on participants during and after the study) **Y/N/U comments**

| **Appendix C: Quality assessment of included studies** | | | | | | | | | | | | | | | | | | | |
| --- | --- | --- | --- | --- | --- | --- | --- | --- | --- | --- | --- | --- | --- | --- | --- | --- | --- | --- | --- |
| *Study* | 1 | 2 | 3 | 4 | 5 | 6 | 7 | 8 | 9 | 10 | 11 | 12 | 13 | 14 | Total | Quality assessment | Risk of bias | No unclear items | |
| Bacchus et al (2003) | + | + | + | + | - | - | N.R | + | + | + | + | + | + | + | 12/14 | A | Attrition domain | | 1 |
| Bates et al (2001) | + | + | + | + | - | - | N.R | + | + | + | + | + | + | + | 12/14 | A | Attrition domain | | 1 |
| Battalgia et al (2003) | + | + | + | + | - | N.R | N.R | N.R | + | + | + | + | + | + | 11/14 | B | Attrition and performance bias | | 3 |
| Belknap & Sayeed (2003) | + | + | + | - | - | N.R | N.R | N.R | + | - | + | + | + | + | 9/14 | D | Selection attrition performance & analysis domain | | 3 |
| Bradbury Jones et al (2011) | + | + | + | + | - | - | N.R | + | + | + | + | + | + | + | 12/14 | A | Attrition domain | | 1 |
| Chang et al (2005a) | + | + | + | + | - | N.R | N.R | + | + | + | + | + | + | + | 12/14 | A | Attrition domain | | 2 |
| Chang et al (2005b) | + | + | + | + | - | - | N.R | N.R | + | + | + | + | + | + | 11/14 | B | Attrition domain | | 2 |
| Damra et al.  (2015) | + | + | - | + | - | - | N.R | N.R | - | + | + | + | + | + | 11/14 | B | Attrition & performance domain | | 2 |
| Dienemann et al.  (2005) | + | + | + | + | - | N.R | - | + | + | + | + | + | + | + | 12/14 | A | Attrition domain | | 1 |
| Gerbert et al. (1999) | + | + | + | + | - | + | N.R | + | + | + | + | + | + | + | 13/14 | A | Attrition domain | | 1 |
| Gerbert et al.  (1997) | + | + | + | + | - | + | N.R | N.R | + | + | + | N.R | + | + | 11/14 | C | Attrition, performance & data analysis domains | | 3 |
| Hathaway et al.  (2002 | + | + | + | + | - | N.R | N.R | + | + | + | + | + | + | N.R | 11/14 | B | Attrition & ethical domain | | 3 |
| Hegarty & Taft.  (2001) | + | + | + | + | - | - | N.R | N.R | + | + | + | + | + | + | 11/14 | B | Attrition & performance domain | | 2 |
| Humphreys et al.  (2003) | + | + | + | + | - | N.R | N.R | - | + | - | + | - | + | - | 8/14 | D | Attrition, performance, data analysis & ethical domain | | 2 |
| Keeling & Fisher  (2015) | + | + | + | + | - | - | N.R | + | + | + | + | + | + | + | 12/14 | A | Attrition domain | | 1 |
| Kelly  (2006) | + | + | + | + | - | N.R | N.R | + | + | + | + | + | + | + | 12/14 | A | Attrition domain | | 2 |
| Liebschutz et al.  (2008) | + | + | + | + | - | - | N.R | N.R | + | + | + | + | + | + | 11/14 | B | Attribution & performance domain | | 2 |
| Lutenbacher et al.  (2003) | + | + | + | + | + | + | N.R | + | + | + | + | + | + | + | 12/14 | A | Attrition domain | | 1 |
| Lutz.  (2005) | + | + | + | + | - | N.R | N.R | + | + | + | + | + | + | + | 12/14 | A | Attrition domain | | 2 |
| Mayer. (2000) | + | + | + | + | - | N.R | N.R | N.R | + | + | + | N.R | + | N.R | 9/14 | D | Attrition, performance, data analysis & ethical domain | | 5 |
| McCauley et al.  (1998) | + | + | + | + | - | N.R | N.R | N.R | + | + | + | + | + | + | 11/14 | B | Attrition & performance domain | | 3 |
| Narula et al.  (2012) | + | + | + | - | - | - | N.R | - | + | + | + | + | + | + | 10/14 | C | Selection, attrition & performance domain | | 1 |
| Nicolaidis et al.  (2008) | + | + | + | + | - | - | N.R | N.R | + | + | + | + | + | + | 11/14 | B | Attrition & performance domain | | 2 |
| Othman et al.  (2014) | + | + | + | - | - | - | N.R | N.R | + | + | + | + | + | + | 10/14 | C | Selection, Attrition & performance domain | | 2 |
| Peckover.  (2003) | + | + | + | + | - | N.R | N.R | N.R | + | + | + | - | + | + | 10/14 | C | Attrition, performance & data analysis domains. | | 3 |
| Reisenhofer & Seibold  (2013) | + | + | + | + | - | - | N.R | N.R | + | + | + | N.R | + | + | 10/14 | C | Attrition, performance & data analysis domains | | 3 |
| Rishal et al. (2016) | + | + | + | + | - | - | N.R | + | + | + | + | + | + | + | 12/14 | A | Attrition domain | | 1 |
| Rodriguez et al.  (1996) | + | + | + | + | - | - | - | + | + | + | + | + | + | + | 12/14 | A | Attrition domain | | 0 |
| Rose et al.  (2011) | + | + | + | + | - | - | N.R | N.R | + | + | + | + | + | + | 11/14 | B | Attrition & performance domain | | 2 |
| Salmon et al.  (2015) | + | + | + | - | - | N.R | - | + | + | + | + | + | + | + | 11/14 | B | Sampling & attrition domain | | 1 |
| Spangaro et al.  (2016a) | + | + | + | + | - | N.R | N.R | + | + | + | + | + | + | + | 12/14 | A | Attrition domain | | 2 |
| Spangaro et al.  (2016b) | + | + | + | + | - | - | N.R | + | + | + | + | + | + | + | 12/14 | A | Attrition domain | | 1 |
| Spangaro et al.  (2011) | + | + | + | + | - | - | N.R | + | + | + | + | + | + | + | 12/14 | A | Attrition domain | | 1 |
| Wallin Lundell et al.(2017) | + | + | + | - | - | N.R | N.R | N.R | + | + | + | + | + | + | 10/14 | C | Selection, attrition & performance bias domains | | 3 |
| Wong et al. (2008) | + | + | + | + | - | - | N.R | + | + | + | + | + | + | + | 12/14 | A | Attrition bias | | 1 |
| Yam (2000) | + | + | + | + | - | N.R | N.R | N.R | + | + | + | + | + | + | 11/14 | B | Attrition & performance domain | | 3 |
| Zink et al.(2004) | + | + | + | + | - | N.R | + | N.R | + | + | + | + | + | + | 12/14 | B | Attrition & performance domain | | 2 |

*Note:* N.R = Not reported. Score of D on quality assessment = study excluded. Additionally, the quality assessment in terms of sampling was defined from Guest, Bunce and Johnson (2006) who proposed that saturation often occurs around 12 participants for a homogenous group. When looking at attrition bias we looked at whether there were any participants that dropped out of the study. In this case it is positive for the quality of the study if the question was answered with no, therefore we counted a no for this question positively in the total score and quality assessment.
